# Myocardial contractility recovery following acute pressure unloading after transcatheter aortic valve intervention (TAVI) in patients with severe aortic stenosis and different left ventricular geometry: a multilayer longitudinal strain echocardiographicanalysis

**DOI:** 10.1007/s10554-020-02074-2

**Published:** 2020-11-30

**Authors:** Sara Cimino, Sara Monosilio, Federico Luongo, Matteo Neccia, Lucia Ilaria Birtolo, Nicolò Salvi, Domenico Filomena, Massimo Mancone, Francesco Fedele, Luciano Agati, Viviana Maestrini

**Affiliations:** Department of Cardiovascular, Respiratory, Nephrological, Anesthesiological and Geriatric, Science “Sapienza” University of Rome, Policlinico Umberto I, Rome Viale del Policlinico 155, 00161 Roma, Italy

**Keywords:** Strain echocardiography, Aortic stenosis, TAVI

## Abstract

Aim of the present study was to describe the left ventricular longitudinal strain (LS) in all myocardial layers in patients with severe aortic stenosis (AS), preserved left ventricular ejection fraction (LVEF) in different LV geometry and to compare LS analysis before and early after acute LV unloading provided by transcatheter aortic valve implantation (TAVI). 68 patients were enrolled. LS was measured from the endocardial layer (Endo-LS), epicardial layer (Epi-LS) and full thickness of myocardium (Transmural-LS) before and after TAVI. Patients were divided in two groups accordingly with relative wall thickness (RWT): concentric LV hypertrophy (cLVH) vs eccentric LV hypertrophy (eLVH). Less impaired values of LS at baseline were observed, in all layers, in patients with cLVHas compared to patients with eLVH (Endo-LS was − 13.2 ± 2 vs − 11.1±3 %, p = 0.041; Epi-LS was − 11.8 ± 1.8 vs − 9.9 ± 3 %, p = 0.043; Transmural-LS was − 12.3 ± 1.8 vs − 10.49 ± 3.3 %, p = 0.02, respectively). A significant improvement in endocardial LS (Endo-LS) after TAVI was detected only in cLVH(− 13 ± 2 vs − 14 ± 2, p = 0.011). Our findings documented that concentric LVH had better basal strain function and showed a better myocardial recovery after TAVI compared to eLVH.

## Background

In patients with aortic stenosis (AS), left ventricular hypertrophy (LVH) is considered to be an adaptive response to the increased afterload [1, 2]. LVH reduces systolic wall stress and helps to preserve LV ejection fraction (LVEF), but it may lead to some long term adverse consequences such as myocardial fibrosis, diastolic filling impairment and finally LV dysfunction [3]. Different types of LVH have been previously described. Gaasch and Zile [4], identified four groups of LV geometry, based on the estimation of LV mass and LV relative wall thickness (RWT). LVH by echocardiography was defined as an increase of the LV mass index > 95 g/m^2^ in women and > 115 g/m^2^ in men. Concentric LVH (cLVH) was characterized by a RWT ≥ 0.42, while eccentric LVH (eLVH) by a RWT < 0.42. These myocardial changes are progressive and associated with symptoms’ onset and prognosis worsening. More severe LVH and, specifically, eLVH pattern seems to be associated with poor prognosis after aortic valve replacement (AVR) [5]. Current guidelines recommend aortic valve replacement for patients with preserved LVEF only at symptoms’ onset, remaining at this stage the main determinant of poor prognosis [6]. However, LVEF has several well know limitation. Longitudinal global strain (LGS) is more sensible to assess subclinical changes in LV function even in patients with preserved LVEF [7]. More recently, multilayer longitudinal strain (LS) has emerged as an innovative tool for a more detailed evaluation of LV mechanics providing a separate analysis in different myocardial layers. This is particularly useful in cardiac disease with expected involvement of sub-endocardial layer (significant coronary artery disease or pressure overload) [4, 8]. Multilayer strain analysis in AS revealed that LS may be impaired in all myocardial layers (sub-endocardial, sub-epicardial and transmural), independently from LV shape [9]. Transcatheter aortic valve implantation (TAVI) is an effective therapeutic option for severe AS, with a significant prognostic impact and an association with cardiac performance improvement over time [10]. However, acute myocardial contractility response after afterload removal in different LV geometry has been not exhaustively described.

Aim of the present study was to examine the acute effect of TAVI in terms of pressure unloading, on LV mechanics using multilayer LS by 2D speckle-tracking echocardiography (ST-E) in different patterns of LV geometry to better understand the subset of AS patients that have potentially more benefits from LV unloading.

## Methods

We enrolled 68 patients (mean age 82 ± 5 years, 34% male, 43% with previous history of coronary artery disease) with severe symptomatic AS and preserved LVEF, scheduled for TAVI. Inclusion criteria were: (a) severe AS defined by an aortic valve area ≤ 0.6 cm^2^/m^2^ by echocardiography, accordingly with current guidelines [6], (b) preserved LVEF (≥ 50% as calculated by 3D echocardiography (Dinamic Heart Model by Philips). Considering these parameters, patients with paradoxical low-flow low-gradient (PLFLG) AS were included.

**Fig. 1:**
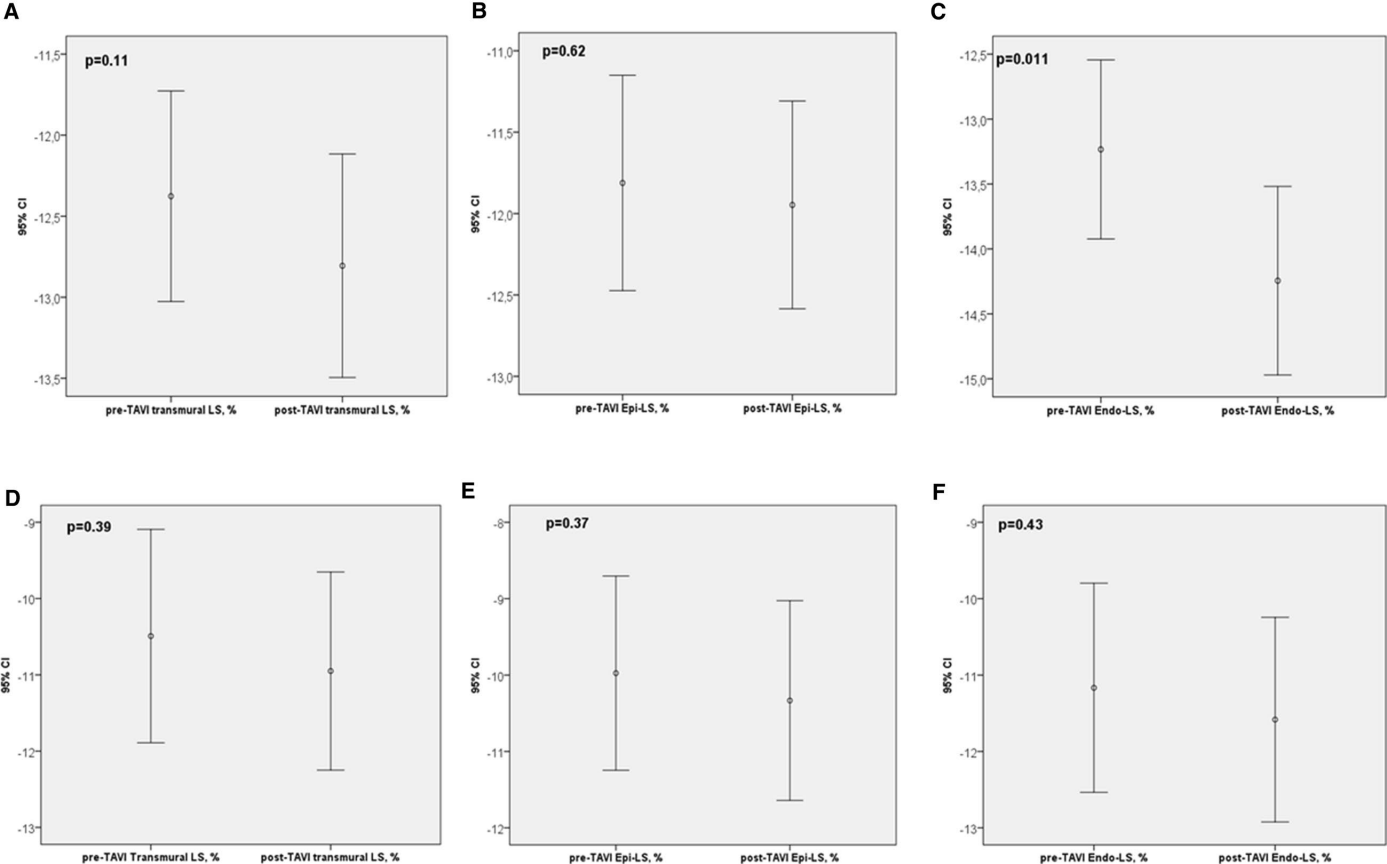
Comprehensive echocardiographic evaluation of aortic stenosis **Panel A** relative wall thickness (RWT) evaluation; **Panel B** aortic valve area evaluation including both planimetric measurements and continuity equation; **Panel C** global longitudinal strain; **Panel D** tissue Doppler imaging; **Panel E** left ventricular ejection fraction (LVEF) in 3D Echo

All patients underwent 2D and 3D echocardiography at baseline and 5 ± 2 days after TAVI. LS by ST-E was measured from the endocardial layer (Endo-LS), epicardial layer (Epi-LS) and full thickness of myocardium (Transmural-LS) before and after the procedure. ST-E analysis was performed with QLab software v.13 by Philips. Analysis included the collection of clinical characteristics and 2D/3D echocardiographic features, such as LV volumes indexed for body surface area (BSA), and ejection fraction (LVEF), LV mass, LV diastolic function, right ventricular (RV) dimension and function and RV-LS. Patients with significant aortic regurgitation or mitral valve disease, pace-maker (PMK) rhythm and/or left bundle branch block (LBBB), unstable coronary artery disease (CAD) were excluded to avoid confounders. Patients with poor acoustic window and uncontrolled heart rate were excluded as contraindications to perform ST analysis. All patients fulfilled LVH hypertrophy criteria by LV mass index. Patients were divided in two groups, accordingly to RWT measurement. cLVH was more represented than eLVH in our study population (46 patients, 68%, vs. 22 patients, 32%). All clinical and demographic characteristics were collected, including stable CAD. The Ethic committee of “Policlinico Umberto I Hospital- Sapienza, University of Rome” approved the present study, and all patients provided written informed consent. Comprehensive echocardiographic evaluation is showed in Fig. 1.

## TAVI procedure

TAVI was performed using a standard trans-femoral or -subclavian technique. Both balloon-expandable and self-expandable valves were used for this purpose. Patients with stable CAD and significant stenosis (> 70%) underwent revascularization with percutaneous angioplasty and drug-eluting stent implantation at least one month before the procedure. A self-expandable valve was implanted in 40 patients (60%), while a balloon-expandable valve was implanted in 28 patients (40%).

### Statistical analysis

Statistical analysis was performed using SPSS software v.23. Continuous variables were reported as mean ± standard deviation, and were compared using Student’s t test or the Mann-Whitney rank sum test for unpaired and paired comparisons, as appropriate. Categorical variables were reported as percentages of individuals. The χ2 test or Fisher’s exact test were used to compare qualitative variables. Differences were considered statistically significant when p < 0.05.

## Results

**Fig. 2:**
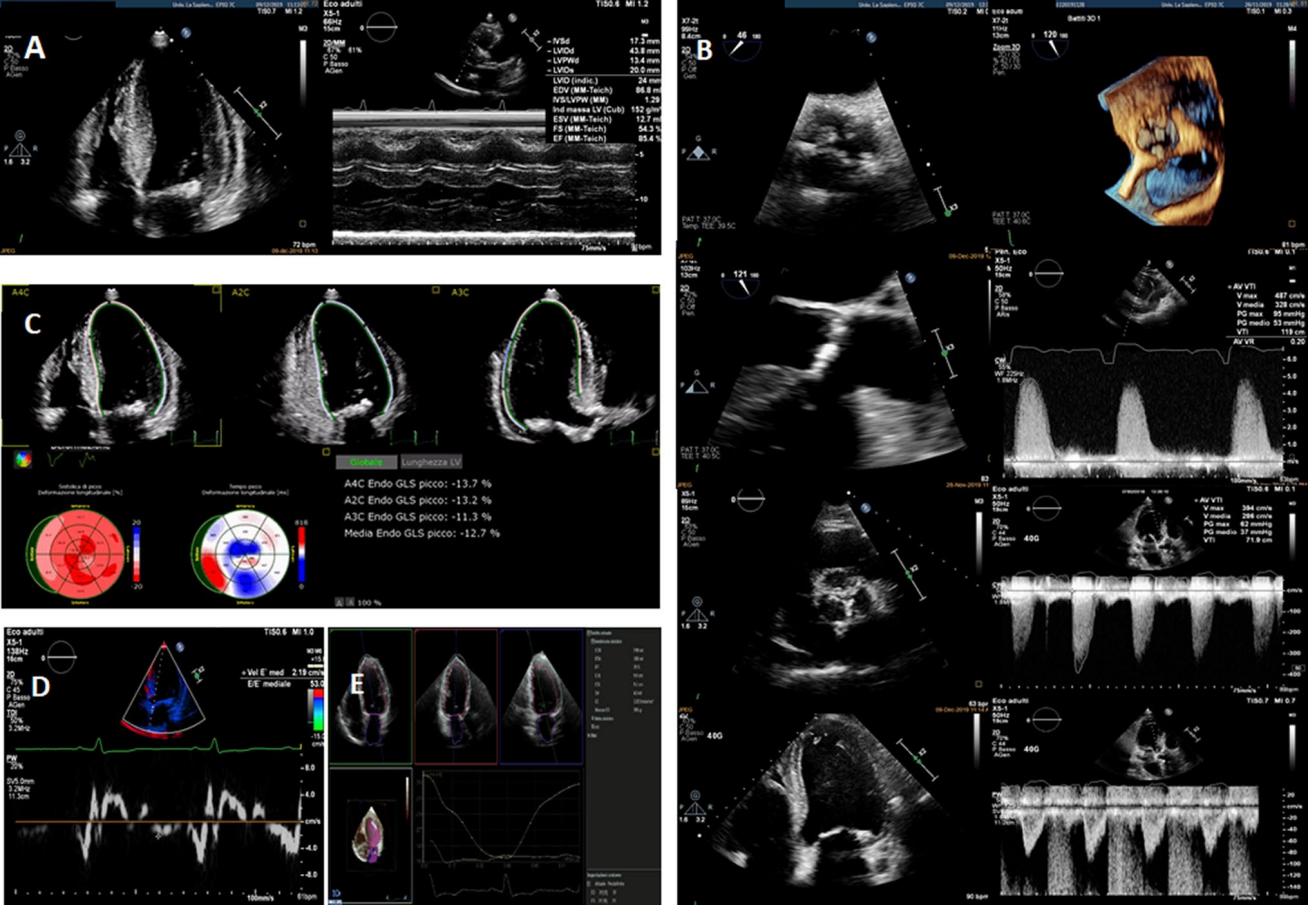
The top row shows differences in transmural (**a**), epicardial (**b**) and subendocardial (**c**) strain before and after TAVI, in cLVH patients. The bottom row **shows** differences in transmural (**e**), epicardial (**f**) and subendocardial (**g**) strain before and after TAVI, in eLVH patients

No significant differences in baseline clinical characteristics were found between the two groups, including the presence of stable CAD, as showed in Table 1. In Table 2 are reported standard echocardiographic characteristics, while Table 3; Fig. 2 show ST-E analysis before and after TAVI. Patients with cLVH were more likely to have smaller LV end-diastolic volume, although no significant (respectively, LVEDV/i 55 ± 15 ml/m^2^ vs. 62 ± 14, p = 0.066) and higher values of LS at baseline in all layers (respectively Endo-LS was − 13.2 ± 2 vs. − 11.1 ± 3%, p = 0.041; Epi-LS was − 11.8 ± 1.8 vs. − 9.9 ± 3%, p = 0.043; Transmural-LS was −  12.3 ± 1.8 vs. -− 0.49 ± 3.3%, p = 0.02). Valvulo-arterial impedance (Zva) was similar in the two groups. After TAVI, a significant improvement in Endo-LS was observed only in cLVH group (− 13 ± 2 vs. − 4 ± 2, p = 0.011). No significant improvements were recorded in eLVH patients after TAVI. Standard echocardiographic parameters didn’t show any significant amelioration immediately after TAVI. No differences in type of valve implanted were found in the two groups.

**Table 1:** Clinical and demographic characteristics

Parameter	cLVH 46 pts (68%)	eLVH 22 pts (32%)	p value
Age, y.o.	82 ± 3	83 ± 5	0.21
Male sex, n (%)	14 (30)	11 (50)	0.31
STS Score	3 ± 0.8	4 ± 1.1	0.08
EuroScore II	4.7 ± 2	4.8 ± 3	0.93
Hypertension, n (%)	37 (80)	18 (81)	0.44
Diabetes, n (%)	11 (25)	5 (25)	0.48
Dyslipidemia, n (%)	23 (50)	14 (63)	0.68
CRF (stage III-IV), n (%)	5 (10)	2 (10)	0.99
CAD, n (%)	18 (40)	9 (40)	0.22

*CAD* coronary artery disease, *CRF* chronic renal failure

**Table 2:** Echocardiographic characteristics in patients with aortic stenosis presenting different LV geometry

Parameters	cLVH 46 pts (68%)	eLVH 22 pts (32%)	p
Before TAVI			
Ao Vmax, m/sec	4.29 ± 0.7	4.12 ± 0.5	0.56
Ao MPG, mmHg	49 ± 15	46 ± 10	0.41
Ao PPG,mmHg	70 ± 22	70 ± 17	0.25
AVA, cm^2^	0.8 ± 0.18	0.7 ± 0.3	0.60
AVA/i, cm^2^/m^2^	0.39 ± 0.12	0.38 ± 0.07	0.63
Zva, mmHg*m^2^/ml	4.1 ± 1.3	4.4 ± 1.1	0.46
PLFLG, n (%)	8 (17)	2 (10)	0.23
LVEDV/i, ml/m^2^	55 ± 15	62 ± 14	0.06
LVESV/i, ml/m^2^	24 ± 6	28 ± 9	0.14
LVEF, %	55 ± 5	56 ± 6	0.99
SV/i, ml/m^2^	41 ± 11	41 ± 10	0.82
LV mass, g/m^2^	158 ± 35	166 ± 54	0.15
LAVi, ml/m^2^	48 ± 19	55 ± 21	0.15
E/A	0.79 ± 0.3	0.93 ± 0.5	0.25
E/e’	12 ± 8	15 ± 4	0.08
TTG, mmHg	29 ± 11	32 ± 10	0.45
TAPSE, mm	22 ± 3	20 ± 2	0.1
RV LS, %	− 18 ± 5	− 16 ± 4	0.36
After TAVI			
LVEDV/i, ml/m^2^	56 ± 14	63 ± 13	0.07
LVESV/i, ml/m^2^	23 ± 5	27 ± 8	0.19
LVEF, %	55 ± 6	56 ± 7	0.99
LAVi, ml/m^2^	47 ± 18	56 ± 22	0.25
E/A	0.8 ± 0.2	0.9 ± 0.6	0.32
E/e’	11 ± 9	16 ± 4	0.09
TTG, mmHg	24 ± 11	34 ± 11	0.48
TAPSE, mm	21 ± 4	20 ± 3	0.99
Self-expandable valve, n (%)	28 (60%)	12 (54%)	0.8
Balloon-expandable valve, n (%)	18 (40%)	10 (46%)	0.8

*Ao* aortic, *AVA *aortic valve area, *cLVH* concentric left ventricular hypertrophy, *eLVH* eccentric left ventricular hypertrophy, *LAV* left atrial volume, *LVEDV* left ventricular end-diastolic volume, *LVESV* left ventricular end-systolic volume, *LS* longitudinal strain, *LVEF* left ventricular ejection fraction,*MPG*mean pressure gradient, *PLFLG* paradoxical low flow low-gradient, *PPG* peak pressure gradient, *RV* right ventricle, *TAPSE* tricuspidal annular systolic escursion, *TTG* trans-tricuspidal gradient, *ZVA*Valvulo-arterial impedance

**Table 3:** Multilayer LV strain analysis before and after TAVI

Parameters	cLVH 46 pts (68%)	eLVH 22 pts (32%)	*p*
Before TAVI			
Transmural-LS, %	− 12.3 ± 1.8	− 10.49 ± 3.3	***0.02***
Epi-LS, %	− 11.8 ± 1.8	− 9.9 ± 3	***0.043***
Endo-LS, %	− 13 ± 2	− 11 ± 3.2	***0.041***
LS gradient, %	− 1.4 ± 0.7	− 1.2 ± 0.6	0.08
After TAVI			
Transmural-LS, %	− 12.8 ± 1.9	− 10.8 ± 3	***0.045***
Epi-LS, %	− 11.9 ± 1.9	− 10.3 ± 3	***0.48***
Endo-LS, %	− 14 ± 2	− 11.5 ± 3.17	***0.003***
LS gradient, %	1.6 ± 0.7	1.12 ± 0.7	0.06

Bold values represent statistically significant values

*Endo* endocardial, *Ep* epicardial, *LS* longitudinal strain, other abbreviations as above

## Discussion

The present study showed (a) the presence of less impaired LS values at baseline, in all layers, in patients with cLVH than in patients with eLVH; (b) a significant improvement of Endo-LS early after TAVI, only in cLVH.

LVH in AS occurs as a response to pressure overload and cLVH pattern is more frequently observed than eLVH, particularly when valve regurgitation and LV systolic dysfunction are excluded [11].The presence of excessive LVH was considered as a negative prognostic marker in patients managed conservatively. Few data are available on the prognostic value of eLVH in this subset of patients, due to the less common presentation [11]. However, since the natural history of AS leads to heart failure (HF), the transition point from the compensatory LVH to decompensation and HF is known to be related with fibrosis and myocardial apoptosis and occurs when the LV fails to counterbalance an increased pressure afterload and is no longer able to maintain forward flow through the stenotic valve. In this stage of the disease, cLVH may shift to eLVH [12].

Accordingly, LS is known to be impaired in AS. It was previously demonstrated that the mechanisms involved in the alteration of LS in AS are (a) the development of LVH and (b) LV fibrosis, which is related with both increased afterload and concomitant CAD [13]. Layer-specific strain pattern is already known to be altered in LVH with different etiologies [14].

Previous studies already explored LS behavior in different myocardial layers after TAVI [15, 16]. Shiino et al. [15] demonstrated a significant LS improvement in all myocardial layers, which was more prominent in sub-endocardial layer, as expected. The same behaviour was observed by Kim et al. [16]who found that LS improvement was greater in patients with higher grade of LVH. In line with these results, our data confirmed that after LV unloading only Endo-LS shows a significant early improvement, but exclusively in one specific subset of patients (cLVH). Our data demonstrated a significant worse baseline LS pattern in eLVH compared to cLVH in all myocardial layers, further supporting a more favorable setting in cLVH. Indeed, eLVH is known to be more frequently associated with further development of heart failure and poor LV adaptation to stress than cLVH [17], and the lack of early Endo-LS recovery in this subset could be a marker of poor prognosis. Our results have to be confirmed in larger longitudinal studies and have to be compared with clinical and echocardiographic follow-up. These preliminary observations have potential implications: advanced valve disease with more severe and eccentric LVH can have less benefits form LV unloading; LV geometry should be taken in account in the decision making process.

## Limitations

The main limitation of the present study is the small sample size that could mask other differences between groups. Follow-up data are not provided, since the study is not powered for MACCE. Finally, there is no control group for comparison.

## Conclusions

Our results demonstrated that patients with severe aortic stenosis and cLVH geometry had a better improvement after TAVI, described by multilayer LS analysis, compared to eLVH. This result highlights the possible role of LV geometry to improve the selection of the stage of valve disease, identifying who could have less benefit from the reduced afterload after aortic replacement.
